# Chronic Obstructive Pulmonary Disease Hospitalization in Spain (2016–2023): Mortality Impact of Comorbidity, Sex-Based Disparities and the Impact of COVID-19

**DOI:** 10.3390/jpm16050255

**Published:** 2026-05-08

**Authors:** Maria Sanchez-McNamara, Maria-Jose Fernandez-Cotarelo, Begoña Perez-de-Paz, Lydia Rodriguez-Romero, Esther Anton-Diaz, Paz Rodriguez-Bolado, Eva Griñan-Fernandez, Victor Moreno, Cesar Henriquez-Camacho

**Affiliations:** 1 Department of Pneumology, Hospital Universitario de Mostoles, 28935 Madrid, Spain; mariasanmac13@gmail.com (M.S.-M.); begoperezdepaz@gmail.com (B.P.-d.-P.); lrromero@salud.madrid.org (L.R.-R.); eantond@salud.madrid.org (E.A.-D.); paz.rodriguezb@salud.madrid.org (P.R.-B.); 2 Department of Internal Medicine, Hospital Universitario de Mostoles, 28935 Madrid, Spain; mariajose.fernandez.cotarelo@urjc.es (M.-J.F.-C.); vmcuerda@salud.madrid.org (V.M.); 3 Department of Medical Specialties and Public Health, Faculty of Health Sciences, Universidad Rey Juan Carlos, Madrid 28922, Spain; evagrinanfernandez@gmail.com; 4 Faculty of Medicine, Universidad Francisco de Vitoria, 28223 Madrid, Spain

**Keywords:** COPD, Charlson comorbidity index, COVID-19

## Abstract

**Background:** COPD remains a leading cause of hospitalization and mortality worldwide. This study aimed to analyze trends in COPD patients in Spain from 2016 to 2023, compare outcomes between patients with COPD as a primary versus secondary diagnosis, and identify factors associated with in-hospital mortality. **Methods**: Retrospective observational study using the Spanish database CMBD. 711.799 patients were analyzed. Demographic characteristics, Charlson Comorbidity Index (CCI), complications, mortality, and hospitalization costs were also evaluated. Multivariate logistic regression was used to identify mortality risk factors. **Results:** The overall hospitalization rate was 20.02 per 1000 admissions. It decreased by 30% during 2020–2021 before rebounding to peak levels in 2023. The proportion of female patients increased from 19.9% (2016) to 26.4% (2023). Patients with COPD as a secondary diagnosis had higher mortality (13% vs. 5.4%, *p* < 0.001), greater comorbidity burden (mean CCI 3.5 vs. 2.8), and higher costs. While overall admissions dropped in 2020, mortality peaked at 11.7%, and the number of patients with extremely severe disease nearly doubled. Independent risk factors for mortality included sepsis, age ≥ 66 years, CCI ≥ 3, and COVID-19. **Conclusions**: Hospitalization involving COPD in Spain showed pandemic-related fluctuations with increasing clinical complexity and increasing female sex. The higher mortality and cost associated with secondary COPD diagnosis highlight the need for comprehensive risk stratification of comorbid conditions and multidisciplinary management of these patients. Early identification of sepsis and CCI scores is essential to improve clinical outcomes in the aging population.

## 1. Introduction

Chronic obstructive pulmonary disease (COPD) is the fourth leading cause of death and the eighth leading cause of poor health (measured by disability-adjusted life years) worldwide [1]. The European Respiratory Society (ERS) and WHO reported that COPD and asthma are the most prevalent chronic respiratory diseases (CRD), with approximately 80% of deaths caused by COPD. The number of patients with COPD is expected to increase by 23% globally by 2050 [2].

COPD is a major public health issue in Spain; it is estimated to affect 12% of the population aged over 40 years. While it is historically more prevalent in men, there is a clear trend toward an increasing incidence among women [3]. Therefore, COPD exacerbations are critical for the patient’s natural evolution of the disease, and hospitalization remains the main driver of expenditure of economic and healthcare resources [4,5,6].

Risk factors for mortality and morbidity have been shown to affect the number, length, and severity of exacerbations [7]. This study aimed to analyze the epidemiological, clinical, and economic characteristics of a broad cohort of patients with COPD, focusing on the J44.0 and J44.1 CIE-10 codes of the Minimum Basic Data Set (MBDS). Utilizing a recent, large-scale Spanish national dataset (2016–2023), this study offers updated insights into pandemic-related fluctuations and provides a unique comparison of outcomes for COPD as a primary versus secondary diagnosis. Our target was to identify factors associated with unfavorable outcomes to contribute to the optimization of clinical and resource strategies.

## 2. Materials and Methods

We conducted a retrospective observational study of patients with COPD based on hospital discharge records from the National Health System between 2016 and 2023. The Ministry of Health provided anonymized data upon request using the minimum basic data set (MBDS). The MBDS includes variables such as sex, date of birth, primary diagnosis, up to 13 secondary diagnoses at discharge, and up to 20 procedures performed during the hospital stay, which were coded according to the International Classification of Diseases, Tenth Revision, Clinical Modification [ICD-10-CM], ICU admission, department responsible for discharge, length of stay (LOS), in-hospital mortality, and costs. The primary diagnosis refers to the condition considered the main cause of hospital admission. Secondary diagnoses were those that coexisted with the main diagnosis either on admission or thereafter during hospitalization. All admissions with a primary or secondary diagnosis of COPD (ICD-10-CM codes J44.0 or J44.1) were registered by a nurse coder based on discharge reports. Our search was based on both primary and secondary diagnoses of the disease.

Comorbidity was assessed by calculating the Charlson Comorbidity Index (CCI) using all diagnoses and procedures recorded at discharge. Specific conditions such as pneumonia, sepsis, pneumococcal infection, heart failure, and Coronavirus Disease (COVID-19) were also recorded. Length of stay (LOS) and in-hospital mortality rates were calculated using admission and discharge dates. The severity of each admitted patient was calculated using the All-Patient Refined Diagnosis-Related Groups (APR-DRG) Severity of Illness (SOI) level. It was assigned one of four levels of severity: low or minor (1), moderate (2), high or major (3), and extreme (4). The SOI uses primary diagnosis, secondary diagnosis (comorbidities and complications), age, procedures performed, and discharge status to calculate the SOI. Admission costs were calculated using the APR-GRD weights.

### 2.1. Statistical Analysis

Statistical analyses were performed using Stata Statistical Software (Release 18). Quantitative variables are expressed as mean ± standard deviation (SD), and qualitative variables are expressed as percentages. The distribution of quantitative variables was determined using the one-sample Kolmogorov–Smirnov test. A bivariable analysis according to year was performed using the chi-square test for linear trends (proportions), analysis of variance (means), and Kruskal–Wallis test (medians) as appropriate. Admission-based hospitalization rates were estimated per 1000 admissions based on data from the Spanish National Institute of Statistics. Multivariate logistic regression analysis was performed for variables that were significant in the univariate analysis and were associated with in-hospital mortality. Adjusted odds ratios (ORs) and 95% confidence intervals (CIs) were calculated. The estimates were expressed as odds ratios (ORs) and 95% confidence intervals (CIs). Statistical significance was set at *p* < 0.05.

### 2.2. Ethics

The study protocol was approved by the Research Ethics Committee of Universidad Rey Juan Carlos (Ref. CEI: 131020256872025). The investigators received anonymized medical data from the MBDS provided by the Spanish Ministry of Health; thus, informed consent was not required.

## 3. Results

### 3.1. Features of Hospitalized COPD Patients During 2016–2023

Table 1 shows a total of 711,799 hospitalizations involving COPD in Spanish hospitals between 2016 and 2023, with a hospitalization rate of 20.02 cases for every 1000 patients. The number of hospitalizations was stable between 2016 and 2019. After this, there was a nearly 30% decrease between 2020 and 2021 during the COVID-19 pandemic compared to pre-pandemic levels, and the numbers rose to the highest in 2023.

**Figure 1:**
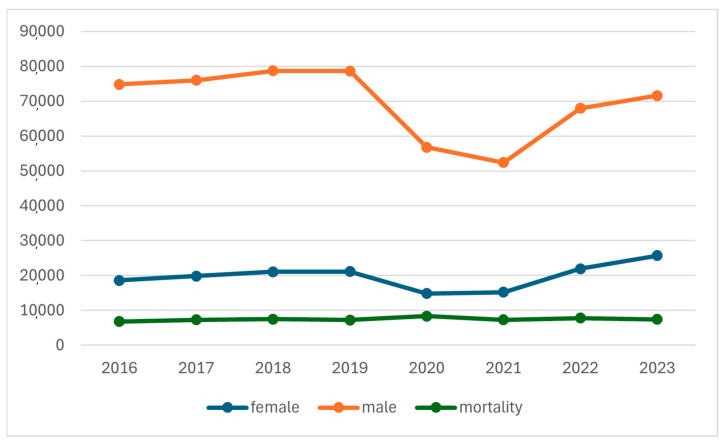
Hospital admissions for COPD in Spain (2016–2023).

The median age was 76 years old. Nevertheless, an increasing proportion of female patients was observed, from 19.9% in 2016 to 26.4% in 2023. The most common age group was ≥66 years (81.2%). (See Figure 1)

Considering the mean Charlson Comorbidity Index, the clinical complexity of the patients slightly increased across the study period; when focusing on an index of ≥3, the proportion of patients increased from 30% in 2016 to 34% in 2023.

During the COVID-19 pandemic, as mentioned previously, the proportion of hospitalizations involving COPD decreased, and there was a decrease in the proportion of COPD as the primary diagnosis. Tendencies in terms of severity also changed in 2020; patients with extreme severity nearly doubled from 8395 in 2019 to 16,187 in 2020. Moreover, the mortality rate increased by up to 11%. Nevertheless, the number of patients requiring ICU hospitalization increased from 703 in 2016 to 894 in 2023. Finally, the costs of hospitalization also increased during the pandemic years and then slightly decreased in 2023.

As for hospitalization complications, respiratory failure showed a steady decreasing tendency, with a difference of 3.6% to 1.3% by the end of the study period.

Heart failure was the most prevalent comorbidity in our study (28.4%).

The mean length of stay was 9 days.

### 3.2. Comparing Mortality in Hospitalized COPD Patients as Primary or Secondary Diagnosis

COPD as a secondary diagnosis showed a significantly higher mortality risk, with an in-hospital mortality rate of 13%, more than double the 5.4% observed in the primary diagnosis group. Furthermore, the secondary diagnosis group required ICU admissions nearly twice as often (9.8% vs. 5.2%), even though the proportion of patients with extremely severe disease was similar in both groups.

The secondary diagnosis group was characterized by a higher proportion of elderly patients (>66 years) and a significantly greater CCI of 3.5 compared with 2.8 for those with a primary COPD diagnosis.

The increased clinical complexity of the secondary diagnosis group translated into a longer mean length of stay (8 days vs. 7 days) and subsequently higher mean cost per hospitalization (3521.52 euros vs. 3036.36 euros) than the primary diagnosis group. (See Supplementary Material Table S1).

### 3.3. Factors Associated with In-Hospital Mortality in Patients with COPD

In the univariate analysis (Table 2), the strongest predictors significantly associated with an increased risk of in-hospital mortality were age ≥66 years, a Charlson Comorbidity Index score of ≥3, ICU admission, diagnosis of sepsis, lung cancer, COVID-19, heart failure, extreme severity, and length of stay >10 days. Pneumonia was not significantly associated with mortality.

The multivariate analysis showed that most factors were statistically significant, although the magnitude of some of the associations was attenuated. Sepsis remained the strongest independent risk factor, along with older age (≥66 years) and Charlson scores of 2 and ≥3. Other significant risk factors included ICU admission, COVID-19, and lung cancer. Pneumonia and heart failure were not significant mortality predictors.

## 4. Discussion

This study provides detailed data from 711,799 hospitalizations involving COPD between 2016 and 2023, corresponding to 10–14% of all hospital admissions during those years. Valuable data were obtained by analyzing various variables, which are discussed below.

Our results confirmed a significant increase in hospitalization of female patients with COPD. This increasing female representation of COPD patients, where female representation rose from 19.9% in 2016 to 26.4% in 2023, aligns with a broader epidemiological trend in Spain and Europe. Data from the Global Burden of Disease across Europe show a similar shift, likely driven by increased tobacco exposure and environmental risk factors in women [8,9]. Furthermore, the 30% reduction in COPD admissions during 2020–2021 is consistent with findings in France and other European countries, where social distancing and mask use likely reduced viral triggers for exacerbations [9]. However, the mortality rate of our study population (8.4% overall) and the significant risk associated with aging emphasize the high risk of elderly patients in Europe [10].

An increase in clinical complexity, as shown by an increase in the Charlson comorbidity index (from 30% to 34% of patients with an index of 3 or above), shows an increase in the burden of disease in hospitalized patients with COPD. In the long term, this finding correlates with worse outcomes and higher expenditure of health care resources.

The year 2020 was marked by the COVID-19 pandemic, which had paradoxical effects on hospitalization with COPD: while total admissions plummeted, the severity and mortality of those admitted increased significantly. In-hospital mortality increased from 7.5% in 2019 to 11.7% in 2020, and the number of patients with extreme severity nearly doubled compared with 2019. This suggests that the most critical cases sought hospital care or that co-infection drastically worsened outcomes. Multivariable analysis identified COVID-19 as a major independent risk factor for death, particularly in those with COPD as a secondary diagnosis. These changes underscore that while preventive measures can reduce exacerbations, no effect on mortality can be observed based on clinical complexity and costs associated with comorbidities during the pandemic.

The increase in hospitalization costs during these years reflects the increase in patient complexity and the need for additional resources to treat COPD exacerbations coexisting with COVID-19. The finding that costs increased during the pandemic (rising by 3177 euros in 2022) despite a drop in admissions is significant and probably related to the extreme severity levels, which nearly doubled in 2020. In a study conducted by Fernandez Villar et al., they found that COPD exacerbations requiring hospitalization showed higher severity and a higher eosinophil count, although mortality at the 90-day point was similar to that in the pre-pandemic years [10,11,12].

Our findings indicate that COPD as a secondary diagnosis featured a high prevalence of specific comorbidities (such as heart failure, pneumonia, COVID-19, and respiratory failure) and showed higher mortality, higher Charlson comorbidity index, longer hospitalization stays, and higher hospitalization costs. These results suggest a worse prognosis for this specific group, a finding consistent with other studies, such as Holguin et al., which reported that approximately 80% of patients with COPD showed this condition as a secondary diagnosis, with higher in-patient mortality [13,14].

Multivariate analysis based on our national cohort reinforced sepsis as the strongest risk factor for in-patient mortality, followed by age > 66 years, lung cancer, and COVID-19. These findings are consistent with and further strengthen the understanding of the main risk factors described in the literature, such as advanced age, comorbidities, and exacerbation severity [7,12,15,16,17,18].

The Charlson comorbidity index is an important risk factor for mortality. Multiple studies have agreed on its prognostic value in COPD, demonstrating that an increase in an index point correlates with a rise in the risk of mortality and readmission of 8–24%. In particular, heart failure and lung cancer were also shown to be important risk factors for mortality [16,19,20,21,22]. Inpatient mortality is consistent with the prognosis predicted in the literature in patients with the coexistence of both conditions, showing a threefold increase in yearly mortality and an increase of up to 61% in long-term mortality [23,24]. Finally, the consistent use of the Charlson index for risk stratification is necessary as a predictor of poor outcomes.

Prevalence of COPD in women has increased progressively, and this is consistent with other studies suggesting a better prognosis for hospitalization [19,25,26]. This requires public health screening programs tailored specifically to women, who now represent an increasing hospital burden. No data on vaccination are available in our study, but the emergence of sepsis and pneumococcal infection as mortality risk factors highlights the need for vaccination policies for patients with COPD.

Since 81.2% of hospitalizations involving COPD are in the elderly, national health policies must focus on geriatric-specific respiratory care and chronic disease management programs by specialists, such as pneumologists, to handle the increasing clinical complexity. Given that hospitalization costs rose during the pandemic and remain high, policy should focus on outpatient exacerbation management and early intervention to reduce the financial strain on the national health system.

The limitations of this study include its retrospective design, the use of databases that can be subject to codifying errors, and the overdiagnosis or underdiagnosis of COPD. Moreover, the lack of details about lung function (spirometry data), specific treatments, smoking status, longitudinal follow-up, and clinical variables limited the interpretation of some findings.

## 5. Conclusions

This study highlights the need for integrative strategies to recognize that hospitalizations involving COPD are of greater clinical complexity and have a rising prevalence among women. Clinical protocols should include high-risk profiles, such as older age, high Charlson comorbidity index, and sepsis. Early identification of comorbidities correctly stratifies patients at high risk of mortality, even as a secondary diagnosis. Implementing early targeted interventions can reduce inpatient mortality. Health care systems should focus on treating COPD patients not only as a primary respiratory disease but also as a complex disease with a multidisciplinary risk-based approach.

## Figures and Tables

**Table 1:** Characteristics of patients with COPD hospitalized in the Spanish National Health System, 2016–2023.

		2016	2017	2018	2019	2020	2021	2022	2023	Total	*p*-Value
Total of cases (%)		93,398 (13)	95,832 (13.5)	99,763 (14)	99,722 (14)	71,623 (10)	67,586 (9.5)	89,906 (12.6)	97,280 (13.7)	711,799	
Total admissions		4,394,783	4,562,937	4,529,568	4,560,676	4,033,467	4,316,803	4,486,174	4,662,877	35,547,285	
COPD per 1000 admissions		21.25	21.00	21.87	21.86	17.76	15.66	20.04	20.86	20.02	
Female sex (%)		18,582 (19.9)	19,861 (20.7)	21,104 (21.2)	21,127 (21.9)	14,869 (20.8)	15,220 (22.5)	21,966 (24.4)	25,690 (26.4)	158,419	<0.001
Mean age (SD)		76 (10)	76 (10)	76 (11)	76 (11)	76 (11)	76 (11)	76 (11)	75 (11)	76 (11)	<0.001
Age group (%)	≤65	16,695 (17.9)	16,715 (17.4)	18,991 (19)	18,192 (18.9)	13,542 (18.9)	13,108 (19.4)	17,332 (19.3)	19,550 (20.1)	134,125 (18.8)	
	≥66	76,703 (82.1)	79,117 (82.6)	80,731 (81)	78,260 (81.1)	58,081 (81.1)	54,478 (80.6)	72,574 (80.7)	77,730 (79.9)	577,674 (81.2)	
COPD as primary diagnosis (%)		58,698 (62.9)	61,027 (63.7)	64,987 (65.2)	62,283 (64.6)	41,853 (58.4)	36,221 (53.6)	48,687 (54.2)	58,535 (60.2)	432,291 (60.7)	<0.001
Charlson categories (%)											<0.001
0–1		45,885 (49)	45,142 (47)	47,073 (47)	43,071 (45)	31,193 (44)	27,859 (41)	39,233 (44)	42,850 (44)	322,306 (45)	
2		19,717 (21)	20,638 (22)	21,277 (21)	21,005 (22)	15,544 (22)	15,327 (23)	19,688 (22)	21,097 (22)	154,293 (22)	
≥3		27,796 (30)	30,052 (31)	31,372 (31)	32,376 (34)	24,886 (35)	24,400 (36)	30,985 (35)	33,333 (34)	235,200 (33)	
Mean Charlson (SD)		2.2 (1.7)	2.2 (1.8)	2.2 (1.8)	2.3 (1.9)	2.4 (2)	2.5 (2)	2.4 (2)	2.4 (2)	2.3 (1.9)	<0.001
Hospital ward	Medical	90,118 (96.5)	92,475 (96.5)	96,372 (96.6)	93,182 (96.6)	68,689 (96)	64,572 (95.5)	86,689 (96.4)	94,026 (96.7)	686,123 (96.4)	<0.001
	Surgical	2126 (2.3)	2168 (2.3)	2194 (2.2)	2132 (2.2)	1805 (2.5)	1795 (2.7)	1976 (2.2)	1927 (2)	16,123 (2.3)	
	ICU	703 (0.8)	778 (0.8)	812 (0.81)	741 (0.77)	829 (1.2)	812 (1.2)	763 (0.9)	894 (0.9)	6332 (0.9)	
	Others	451 (0.5)	411 (0.4)	344 (0.3)	397 (0.4)	300 (0.4)	407 (0.6)	478 (0.5)	433 (0.5)	3221 (0.5)	
Pneumonia (%)		5272 (5.6)	4939 (5.2)	4027 (4)	3905 (4)	2662 (3.7)	2146 (3.2)	2797 (3.1)	3969 (4.1)	29,717 (4.2)	<0.001
Pneumococcal sepsis (%)		76 (0.1)	99 (0.1)	138 (0.1)	162 (0.2)	87 (0.1)	54 (0.1)	117 (0.1)	204 (0.2)	937 (0.1)	<0.001
Sepsis (%)		979 (1.1)	1003 (1.1)	1092 (1.1)	1102 (1.1)	777 (1.1)	729 (1.1)	996 (1.1)	1254 (1.3)	7932 (1.1)	<0.001
COVID-19 (%)		-	-	-	-	3998 (5.6)	7408 (11)	11,631 (13)	5408 (5.6)	28,445 (4)	<0.001
Bronchitis (%)		750 (0.8)	626 (0.7)	779 (0.8)	846 (0.9)	542 (0.8)	351 (0.5)	456 (0.5)	563 (0.6)	4913 (0.7)	<0.001
Pneumococcal pneumonia (%)		462 (0.5)	528 (0.6)	425 (0.4)	523 (0.5)	354 (0.5)	219 (0.3)	353 (0.4)	611 (0.6)	3475 (0.5)	<0.001
Flu (%)		35 (0.04)	37 (0.04)	117 (0.1)	97 (0.1)	76 (0.1)	7 (0.01)	69 (0.1)	98 (0.1)	536 (0.1)	<0.001
Respiratory failure (%)		3321 (3.6)	2689 (2.8)	1943 (2)	1635 (1.7)	978 (1.4)	879 (1.3)	1071 (1.2)	1232 (1.3)	13,748 (1.9)	<0.001
Heart failure (%)		24,235 (26)	25,698 (26.8)	26,974 (27.1)	27,748 (28.8)	20,385 (28.5)	21,546 (31.9)	26,648 (29.6)	29,086 (30)	202,320 (28.4)	<0.001
Severity	Low	7025	5748	6309	5383	3565	3140	6728	7776	45,674	<0.001
	Moderate	29,967	29,093	29,894	28,466	20,281	18,231	24,794	28,197	208,923	
	High	50,106	53,916	55,593	54,121	31,571	30,734	46,764	47,583	370,388	
	Extreme	6165	6928	7875	8395	16,187	15,471	11,587	13,704	86,312	
Mean LOS days (SD)		8.8 (9.1)	8.7 (9.5)	8.8 (9.3)	8.8 (10.2)	9.2 (10.6)	9.6 (11)	9 (10)	8.7 (10.2)	8.9 (10)	<0.001
In-hospital mortality (%)		6804 (7.3)	7297 (7.6)	7483 (7.5)	7245 (7.5)	8380 (11.7)	7303 (10.8)	7800 (8.7)	7428 (7.6)	59,740 (8.4)	<0.001
Mean cost (EUR)		2563.96	2530.76	2541.66	2633.29	3119.36	3079.10	3177.52	3135.10	2826.12	<0.001

Abbreviations: COPD: Chronic Obstructive Pulmonary Disease; SD, standard deviation; ICU: Intensive Care Unit; LOS: Length of stay; COVID-19: Coronavirus disease.

**Table 2:** Multivariate analysis of factors associated with in-hospital mortality of COPD.

		In-Hospital Mortality		
		Primary Diagnosis Univariate Analysis	Secondary Diagnosis Univariate Analysis	Multivariate Analysis
		aOR (95%CI) *p* Value	aOR (95%CI) *p* Value	aOR (95%CI)
Male sex		0.70 (0.67–0.72) <0.001	0.78 (0.76–0.80) <0.001	0.86 (0.84–0.89) <0.001
Age ≥ 66		3.36 (3.17–3.50) <0.001	2.11 (2.03–2.19) <0.001	2.51 (2.43–2.59) <0.001
CCI	≤1 (reference)	1	1	1
	2	2.05 (1.98–2.13) <0.001	1.57 (1.52–1.62) <0.001	1.64 (1.59–1.68) <0.001
	≥3	2.78 (2.70–2.87) <0.001	2.50 (2.43–2.57) <0.001	2.38 (2.33–2.44) <0.001
Hospital ward	Medical	0.53 (0.45–0.63) <0.001	0.55 (0.48–0.62) <0.001	0.52 (0.47–0.58) <0.001
	Surgical	2.39 (1.89–3.03) <0.001	0.58 (0.51–0.66) <0.001	0.80 (0.72–0.90) <0.001
	ICU	26.82 (22.2–32.67) <0.001	13.23 (11.46–15.27) <0.001	16.26 (14.40–18.36) <0.001
COVID-19		-	1.24 (1.19–1.28) <0.001	1.68 (1.62–1.74) <0.001
Pneumonia		-	0.52 (0.49–0.54) <0.001	0.95 (0.91–0.99) 0.041
Lung Cancer		-	2.47 (1.32–4.39) <0.001	3.76 (2.11–6.69) <0.001
Sepsis		-	3.66 (3.49–3.84) <0.001	3.76 (3.57–3.96) <0.001
Heart failure		-	1.27 (1.25–1.30) <0.001	1.00 (0.98–1.03) 0.385
Severity	Low (reference)	1	1	1
	Moderate	2.27 (2.08–2.49) <0.001	0.26(0.21–0.33) <0.001	0.24 (0.19–0.31) <0.001
	High	4.12 (3.78–4.48) <0.001	0.61 (0.49–0.76) <0.001	0.44 (0.34–0.56) <0.001
	Extreme	14.54 (13.34–15.85) <0.001	1.85 (1.48–2.31) <0.001	1.10 (0.86–1.40) 0.431
LOS	>10 days	2.11 (2.05–2.16) <0.001	1.25 (1.22–1.28) <0.001	1.21 (1.19–1.24) <0.001

Abbreviations: ORa, adjusted odds ratio; CCI, Charlson comorbidity index; ICU, intensive care unit; COVID-19, Coronavirus disease; LOS, length of stay.

## Data Availability

The dataset used and analyzed in the current study is available from the corresponding author upon reasonable request.

## Supplementary Materials

The following supporting information can be downloaded at: https://www.mdpi.com/article/10.3390/jpm16050255/s1, Table S1. Comparison of mortality in COPD patients as a primary or secondary diagnosis.
