# Supplementary material for: Chronic Obstructive Pulmonary Disease Hospitalization in Spain (2016–2023): Mortality Impact of Comorbidity, Sex-Based Disparities and the Impact of COVID-19
Source: J Pers Med. 2026 May 8;16(5):255. doi: 10.3390/jpm16050255 (PMC13208161; doi:10.3390/jpm16050255)
Supplement: Supplementary file 1 [file jpm-16-00255-s001.zip › jpm-4242636-supplementary.pdf]

Supplementary Table S1. Comparison of mortality in COPD patients as a primary or secondary diagnosis.

		Primary diagnosis N (%)	Secondary diagnosis N (%)	p-value
Mortality		23362 (5.4)	36378 (13)	<0.001
Total of cases		432291	279508	
Female sex		4084 (18)	6534 (18)	0.236
Mean age (SD)		81 (9)	80 (10)	<0.001
Age group (%)	≤ 65	1710 (7.3)	3419 (9)	
	≥ 66	21652 (93)	32959 (91)	
Charlson means (SD)		2.8 (2.2)	3.5 (2.6)	<0.001
Hospital ward	Medical	21818 (93.4)	30626 (84.2)	<0.001
	Surgical	165 (0.7)	1905 (5.2)	
	ICU	1225 (5.2)	3549 (9.8)	
	Others	154 (0.7)	298 (0.8)	
Severity	Low	570 (2.4)	93 (0.3)	<0.001
	Moderate	3329 (14.3)	5811 (16)	
	High	11931 (51)	18132 (50)	
	Extreme	7532 (32)	12245 (34)	
Median LOS days (IQR)		7 (3-15)	8 (3-16)	<0.001
Mean cost EUR (SD)		3036.36 (878)	3521.52 (1476)	<0.001

Abbreviations: COPD: Chronic Obstructive Pulmonary Disease; SD: standard deviation; ICU: Intensive Care Unit; LOS: Length of stay; COVID-19: Coronavirus disease; IQR: interquartile range.
